# Chronic actinic dermatitis as a diagnostic clue for adult T-cell leukemia/lymphoma

**DOI:** 10.1016/j.jdcr.2024.10.021

**Published:** 2024-11-09

**Authors:** Sakiho Inayoshi, Takuya Inoue, Hiroo Katsuya, Kazunari Sugita

**Affiliations:** Division of Dermatology, Department of Internal Medicine, Faculty of Medicine, Saga University, Saga, Japan

**Keywords:** adult T-cell leukemia/lymphoma, anti-human T cell leukemia virus type-1, chronic actinic dermatitis

## Introduction

Chronic actinic dermatitis (CAD), previously known as actinic reticuloid, is a photosensitivity dermatitis characterized by persistent eczematous lesions, including infiltrative papules and plaque, predominantly appearing in sun-exposed areas. The exact cause of CAD remains elusive.^1^ Commonly affecting elderly males, CAD manifests with abnormal photosensitivity to ultraviolet A (UVA) and/or ultraviolet B (UVB). Although CAD can be associated with photocontact allergy involving various antigens,^2^ the prevalence of CAD in a significant number of HIV patients suggests the involvement of immunological mechanisms in its pathogenesis.^3^ This report highlights a unique case where a CAD patient exhibited symptoms preceding the diagnosis of adult T-cell leukemia/lymphoma (ATLL). Notably, the CAD symptoms resolved upon the initiation of ATLL treatment.

## Case report

**Fig 1:**
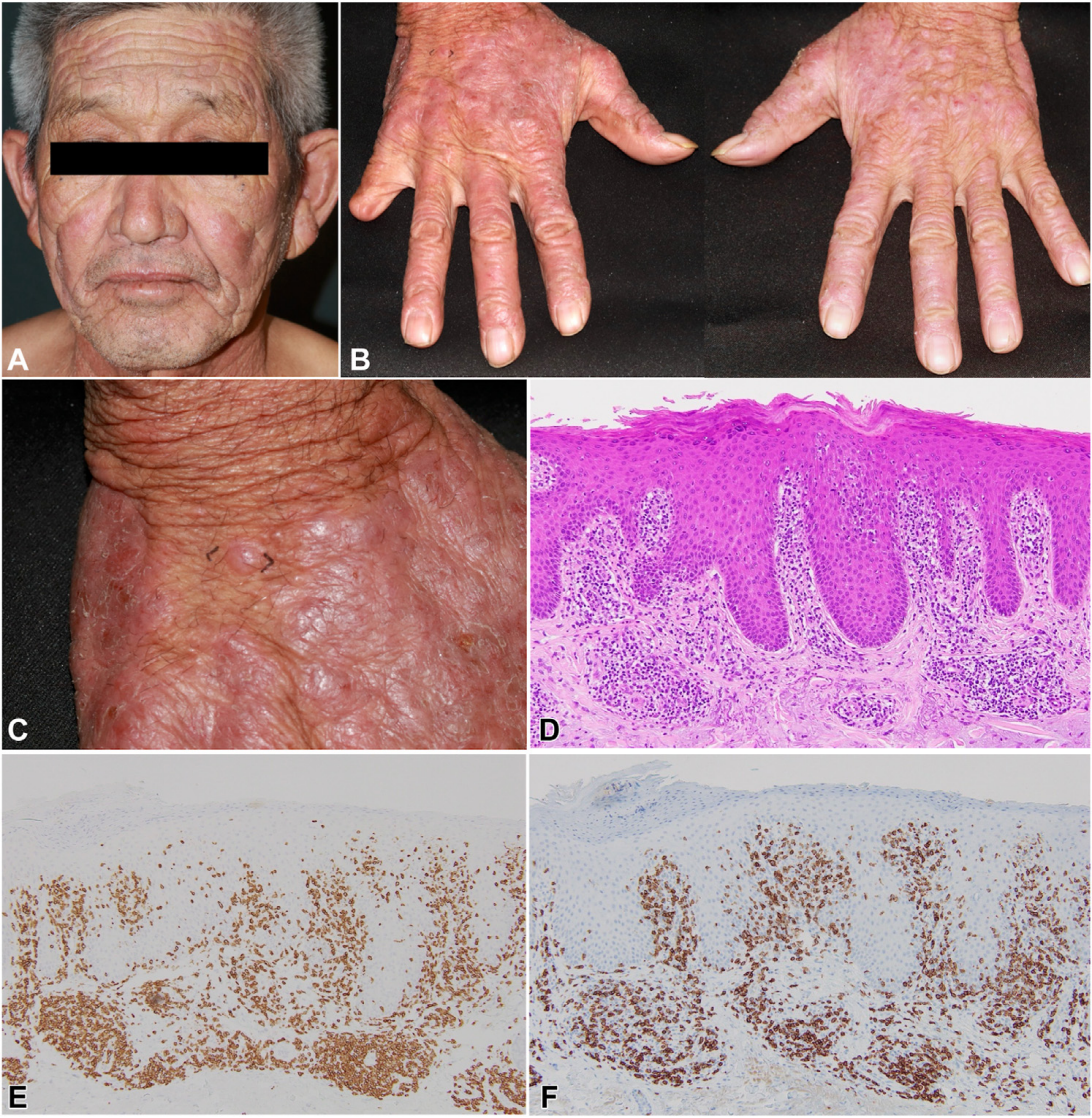
**A** and **B,** Clinical pictures on the face and hands. **C,** A skin biopsy was taken in a spindle shape from the *black-marked* area on the dorsum of the right hand. **D,** Hematoxylin and eosin (H & E) stain, ×200. **E,** CD3 stain, ×200. **F,** CD8 stain, ×200.

A 72-year-old male with a medical history of arteriosclerosis obliterans, diabetes mellitus, and hyperlipidemia was referred to our clinic with a 1-year history of pruritic papules in sun-exposed areas. Upon physical examination, an infiltrative erythema was noted on the face, V-neck area, forehead, and hands (Fig 1, *A* and *B*). A skin biopsy from the red-purple plaque on the dorsum of the right hand (Fig 1, *C*) indicated superficial perivascular dermatitis with hyperkeratosis and parakeratosis, lacking nuclear atypia of infiltrating lymphocytes (Fig 1, *D*). Immunohistochemically, a majority of immune cells tested positive for CD3, predominantly comprising CD8⁺ lymphocytes (Fig 1, *E* and *F*). The patient demonstrated a reduced minimal erythema dose at 20 mJ/cm^2^ and exhibited no erythema upon UVA exposure. Blood tests disclosed elevated levels of lactate dehydrogenase (371 U/I; normal range 124-222 U/I), IL-2 receptor (1597 U/ml; normal range 122-496 U/ml), and a positive anti-human T cell leukemia virus type-1 (HTLV-1) antibody. The CD4/CD8 ratio in peripheral blood decreased to 0.57. Despite these findings, neither palpable enlarged lymph nodes nor abnormalities on computerized tomography (CT) were detected. Based on clinical and histopathologic evidence, a diagnosis of CAD was considered in an HTLV-1 positive patient.

**Fig 2:**
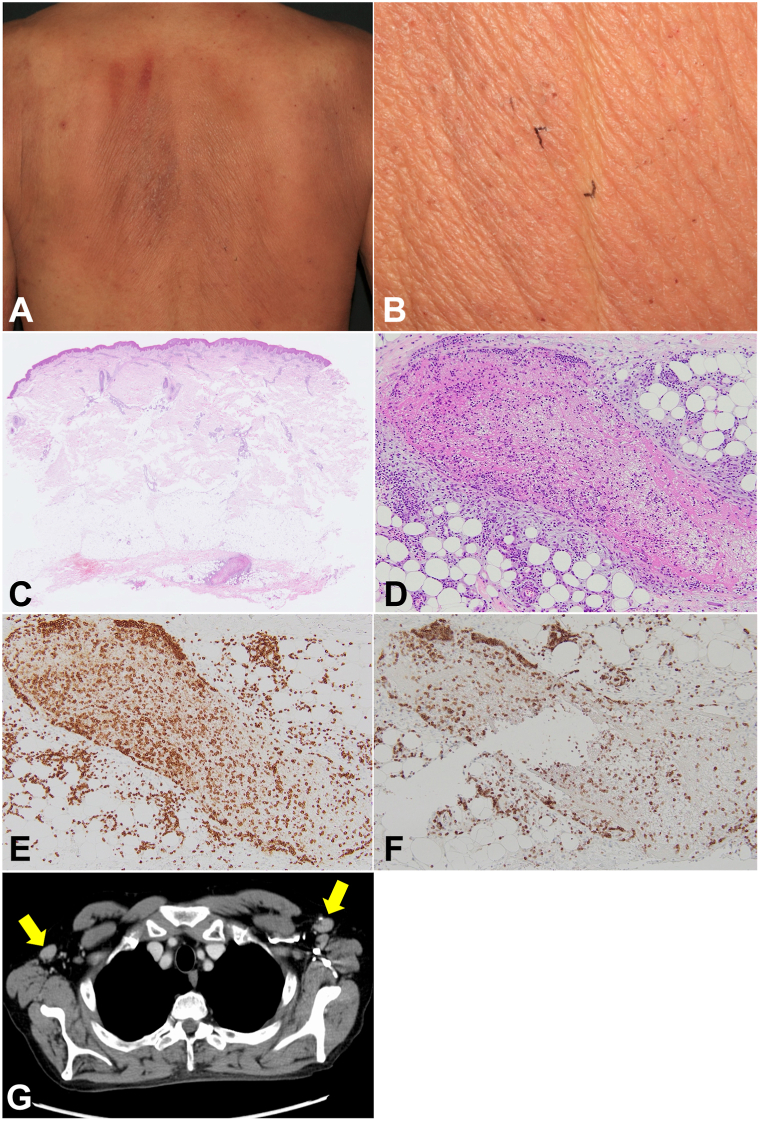
**A,** Clinical picture of erythematous eruption on his back. **B,** High power view on the back. A skin biopsy was performed in a spindle shape from the *black-marked* area on the back. **C,** H & E stain, ×100. **D,** H & E stain, ×200. **E,** CD3 stain, ×200. **F,** CCR4 stain, ×200. **G,** CT image. *Arrows* indicate enlarged axillary lymph nodes. *CT*, Computerized tomography.

Two months later, a follow-up CT revealed enlarged axillary lymph nodes, with positron emission tomography CT confirming accumulation in the same area. Despite the absence of atypical lymphocytes in the peripheral blood, we maintained vigilant monitoring of the patient. Four months after the initial examination, a diffusely spreading erythematous eruption appeared on his back (Fig 2, *A*). A skin biopsy from the back disclosed infiltration of atypical lymphocytes in the subcutaneous tissue (Fig 2, *B*-*D*). Immunohistochemically, a majority of the cells tested positive for CD3, and most CD3^+^cells were found to be CCR4 positive (Fig 2, *E* and *F*). Six months from the initial examination, abnormal lymphocytes emerged in the peripheral blood, coinciding with the CT image revealing the presence of enlarged axillary lymph nodes (Fig 2, *G*). A lymph node biopsy confirmed the involvement of atypical lymphocytes with CCR4 positivity. Subsequently, we diagnosed lymphoma-type of ATLL based on the Shimoyama classification in a patient with CAD. The treatment regimen included CHOP chemotherapy (Cyclophosphamide, Doxorubicin Hydrochloride, Oncovin(Vincristine), and Prednisone), mogamulizumab, and VECP chemotherapy (Vindesine, Etoposide, Carboplatin, and Prednisone). Following these therapies, serum IL-2 receptor levels gradually decreased alongside improvements in CAD lesions and the infiltrative erythema on his back. Notably, the CD4/CD8 ratio in the peripheral blood increased to 0.83 compared to the initial diagnosis. Throughout the course of ATLL treatment, medications for arteriosclerosis obliterans, diabetes mellitus, and hyperlipidemia remained unchanged, and we ruled out the possibility of drug-induced photosensitivity.

## Discussion

ATLL is attributed to HTLV-1 infection, with carriers prevalent in regions such as Africa, the Caribbean, Latin America, and Japan.^4^ Conversely, CAD has predominantly surfaced in HIV-positive patients, with its pathogenesis linked to an imbalance between CD4^+^ and CD8^+^ cells, coupled with a numerical decline in CD4 positive cells.^5^ Notably, in the current case, a diminished CD4/CD8 ratio in peripheral blood was observed, and CD8⁺cells significantly infiltrated the cutaneous localization of CAD, leading to a lichenoid tissue reaction. This suggests that CD8⁺ cells may be activated due to abnormalities in HTLV-1 infected CD4^+^CCR4^+^ cells. During the initial examination, the patient did not manifest enlarged lymph nodes; however, a notably high serum IL-2R level was observed. This implies a potential proliferation of specific ATLL clones within the lymph nodes before the detection of lymph node swelling in CT. Given the rarity of both CAD and ATLL, the simultaneous presence of CAD in ATLL in the present case may be coincidental, but it is more likely to be causally linked.

Recently, it has been reported that HTLV-1 carriers exhibit an expansion of CD8^+^ cells in the peripheral blood and a reduction in the CD4/CD8 ratio before the onset of ATLL.^6^ Additionally, myelopathy associated with HTLV-1 and cutaneous manifestations in HTLV-1-infected patients have been observed to involve reduced CD4/CD8 ratios in peripheral blood and local infiltration of activated CD8^+^ cells.^7^ Consequently, it is conceivable that CAD manifested in this patient against a backdrop of immunologic abnormalities ranging from HTLV-1 carrier status to the onset of ATLL. The correlation between skin symptoms and treatment has not been previously discussed in cases of complicated CAD in ATLL.^8^^,^^9^ Notably, the resolution of CAD after ATLL treatment and an approximately 1.5-fold increase in CD4/CD8 counts compared to the initial diagnosis strongly support in the idea that ATLL is closely linked to the development of CAD.

Based on these findings, we strongly recommend adopting a proactive screening approach for HTLV-1 in individuals with CAD, particularly in areas with a high prevalence of HTLV-1. Recently, reports have indicated that dupilumab, used for treating atopic dermatitis, has been associated with unmasking cutaneous T-cell lymphoma in some patients.^10^ While dupilumab has shown effectiveness in treating intractable CAD, clinicians should exercise caution when prescribing dupilumab to HTLV-1 carriers with CAD, as CAD may precede the development of ATLL, as observed in the present case. Close monitoring is recommended to detect early signs of ATLL in such patients. These strategies can aid in the early detection of HTLV-1 carriers and enhance our comprehension of the complex interplay between HTLV-1, ATLL, and CAD.

## Conflicts of interest

None disclosed.
